# Supplementary material for: 11β-Hydroxysteroid Dehydrogenase Type 1 within Osteoclasts Mediates the Bone Protective Properties of Therapeutic Corticosteroids in Chronic Inflammation
Source: Int J Mol Sci. 2022 Jun 30;23(13):7334. doi: 10.3390/ijms23137334 (PMC9266304; doi:10.3390/ijms23137334)
Supplement: Supplementary file 1 [file ijms-23-07334-s001.zip › ijms-1746201-supplementary.pdf]

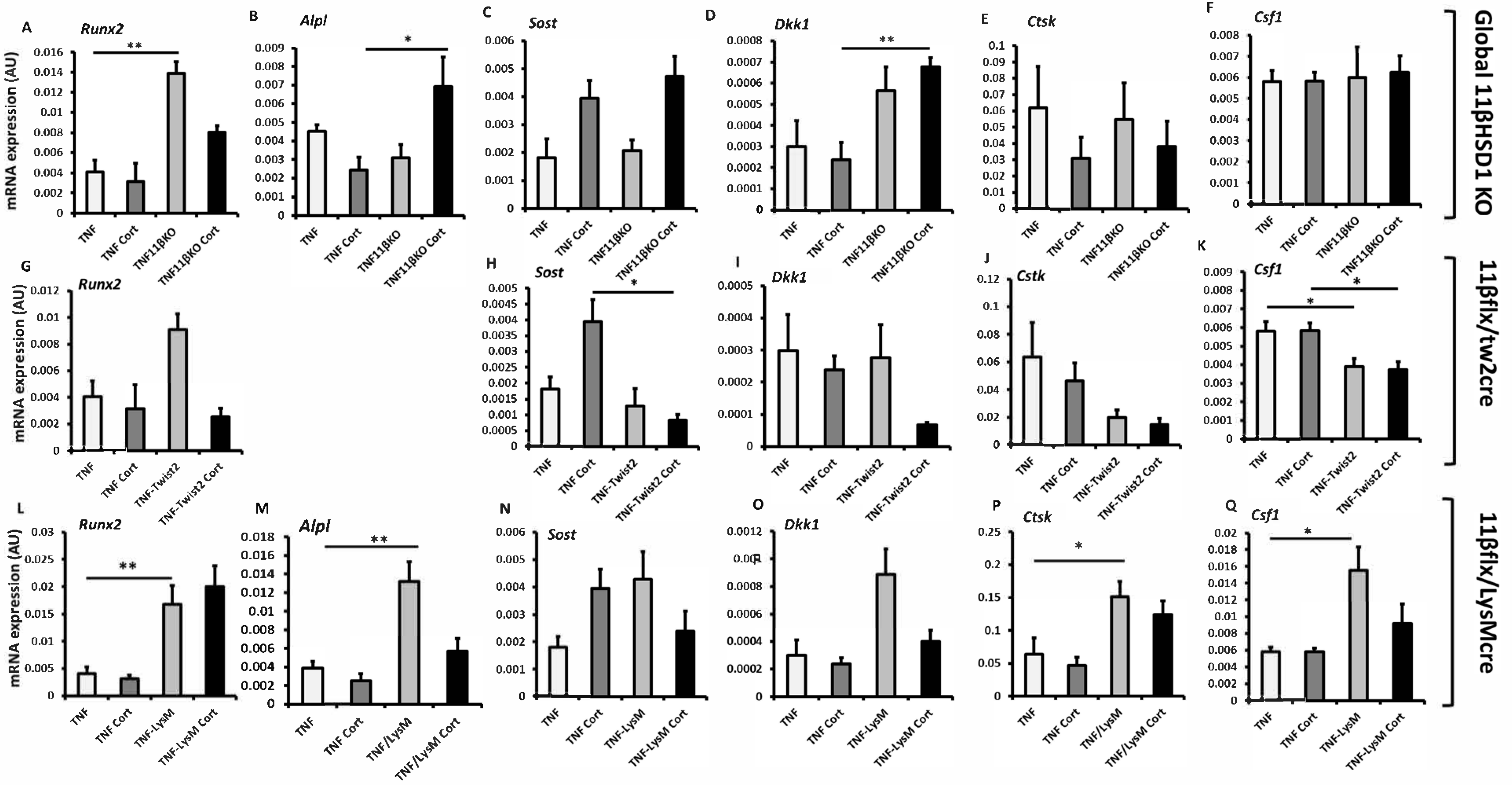

Supplementary Figure S1. (A) mRNA expression (AU) of (A,G,L) Runx2, (B,M) Alpl, (C,H,N) Sost, (D,I,O) Dkk1, (E,J,P) Ctsk, (F,K,Q) Csf1 in the tibias of TNF-tg, TNF-tg$^{11\beta KO}$, TNF-tg$^{11\beta flx/tw2cr}$ *and TNF-tg$^{11\beta flx/LysMcre}$ animals receiving either vehicle or corticosterone (100 µg/mL) in drinking water over 3 weeks determined by either RT-qPCR. Values are expressed as mean ± standard error of six animals, per group. Statistical significance was determined using two-way ANOVA with a Tukey post hoc analysis. * $p<0.05$, ** $p<0.005$.*
